# The Effect of Intolerance of Uncertainty on Indecisiveness in Anxiety and Obsessive‐Compulsive Disorders

**DOI:** 10.1002/jclp.70160

**Published:** 2026-06-05

**Authors:** Helmut Appel, André Mattes, Alexander L. Gerlach

**Affiliations:** ^1^ Institute of Clinical Psychology and Psychotherapy University of Cologne Cologne Germany; ^2^ Department of Individual Differences and Psychological Assessment University of Cologne Cologne Germany

**Keywords:** decision making, indecisiveness, intolerance of uncertainty, transdiagnostic factors

## Abstract

**Objectives:**

Intolerance of uncertainty (IU) describes a dispositional aversion to uncertainty. Recent research has identified IU as a causal contributor to pervasive decision difficulties (indecisiveness). Given that IU and indecisiveness occur across mental disorders, understanding whether IU's causal role in indecisiveness extends to clinical populations is critical. This study therefore tested the effect of IU on indecisiveness in a clinical sample.

**Methods:**

In this pre‐registered experiment, *N* = 154 individuals with formally diagnosed obsessive‐compulsive and/or anxiety disorders were randomly assigned to a condition aimed at either increasing or decreasing IU. Subsequently, participants reported their current levels of (situational) IU and their indecisiveness regarding two personally relevant decisions.

**Results:**

While the manipulation had no main effect on indecisiveness, it influenced situational IU levels, which were positively associated with indecisiveness. Mediation analysis indicated a significant indirect effect of the experimental condition on indecisiveness via situational IU.

**Limitations:**

Compared to preceding research, effect sizes were small and the effect of IU on indecisiveness only manifested itself indirectly. More robust interventions may be necessary to induce more pronounced changes in IU within clinical populations. The reliance on a heterogeneous sample with various diagnoses limits the disorder‐specificity of the findings.

**Conclusions:**

These results tentatively extend the causal role of IU in indecisiveness to mental disorders. The findings suggest IU as a potential approach for addressing clinically relevant indecisiveness if future research corroborates the evidence.

## 1 Introduction

Decisions pervade every day of our lives. In the area of food alone—an admittedly important, but only partial section of our daily decisions—an average of over 200 decisions and micro‐decisions are made per day (Wansink and Sobal 2007). Extrapolating this estimate to all life domains results in an enormous number of choices each day. A mental disorder that has a negative impact on decision‐making ability will therefore likely interfere severely with psychological functioning. The present study focuses on one important aspect influencing decision making, namely uncertainty. More specifically, it investigates how handling uncertainty causally influences decision‐making difficulties in people with anxiety‐related mental disorders.

Dispositional difficulties in coping with uncertainty are referred to as intolerance of uncertainty (IU) and are defined as “an individual's dispositional incapacity to endure the aversive response triggered by the perceived absence of salient, key, or sufficient information, and sustained by the associated perception of uncertainty” (Carleton 2016, p. 31). Individuals with higher IU levels show altered responses to uncertainty across multiple levels (for an overview, see Morriss et al. 2023; Tanovic et al. 2018): neurobiologically (e.g., increased reactivity to uncertainty in the anterior insula, a brain region thought to contribute to anticipatory responses, Gorka et al. 2016); physiologically (e.g., increased electrodermal response in extinction learning involving uncertain threat, Morriss et al. 2016); behaviorally (e.g., increased checking of uncertain stimuli, Wake et al. 2022); and emotionally (e.g., increased negative affect and reduced positive affect in response to uncertainty, Morriss et al. 2023). Together, these findings show that elevated IU is associated with difficulties in effectively dealing with uncertain situations. Decision‐making is a domain that inherently requires the management of uncertainty, as choices often must be made in the face of incomplete or ambiguous information.

In line with this, IU is thought to have negative implications for decision‐making (Koerner et al. 2017; Rassin et al. 2007) and empirical findings support this view: According to behavioral studies, IU is associated with potentially disadvantageous patterns in decision‐making (Carleton et al. 2016). For example, in a gambling task, Luhmann et al. (2011) found IU was linked to a preference for immediately available but smaller and less probable rewards over larger, more probable rewards that were only available after a delay, likely to avoid waiting time and the associated uncertainty. IU is also connected to other decision‐related phenomena such as reduced confidence in decisions (Jensen et al. 2014), or increased attempts to reduce decisional uncertainty (Jacoby et al. 2014).

IU is accordingly considered a risk factor for indecisiveness (Rassin 2007). Indecisiveness is defined as a “trait‐like difficulty making decisions across time and situations” (Lauderdale and Oakes 2021, p. 256). This difficulty manifests on multiple levels, encompassing affective experiences (e.g., feeling conflicted, van de Jong‐Meyer 2009), cognitive aspects like post‐decisional doubts (Germeijs and De Boeck 2002), and behavioral expressions such as decision delay (Frost and Shows 1993) or avoidance (Rassin and Muris 2005b). Consistent with this, indecisiveness has been linked to impairments in everyday life, including procrastination on important tasks (Ferrari 1994) or difficulties committing to academic goals (Germeijs and Verschueren 2010).

Empirically, indecisiveness and IU are consistently associated, both at the trait level (e.g., Koerner et al. 2017) and in day‐to‐day decisions (Appel et al. 2024). However, they represent distinct constructs. Whereas IU reflects a general aversion to uncertainty, indecisiveness is specific to decision‐making. Because decisions inherently involve uncertainty, IU may act as a higher‐order predisposition that, in decision‐making contexts, contributes to the subjective and behavioral difficulties characteristic of indecisiveness (Koerner et al. 2017; Rassin 2007).

Directly supporting IU as a predisposing antecedent of indecisiveness, Appel and Gerlach (2025a) experimentally manipulated IU in a study (*N* = 301) using a validated paradigm (Britton and Davey 2014). More specifically, participants read a vignette about a person struggling either with excessive sensitivity to uncertainty (Decrease IU condition) or with insufficient responsiveness to uncertainty (Increase IU condition). Depending on the condition, participants then were asked to generate advice encouraging either greater tolerance of uncertainty (Decrease IU condition) or stronger negative reactions to uncertainty (Increase IU condition), alongside a neutral control condition unrelated to uncertainty. Following the manipulation, participants reported their situational IU (manipulation check). More importantly, they reported their situational indecisiveness (dependent variable) regarding both a personally relevant decision and a standardized lottery choice.

The manipulation successfully altered situational IU, such that situational IU was significantly higher in the Increase condition compared to both the control condition and the Decrease condition. The Decrease condition did not differ significantly from the control condition. Examining the key contrast between the Increase and Decrease conditions (differences involving the control condition were less consistent) revealed a small‐to‐medium effect (*d* = 0.46) on situational IU and, more importantly, a medium‐sized multivariate effect on both indecisiveness measures (partial *η* ^2^ = 0.07), with higher levels in the Increase condition compared to the Decrease condition. Additional exploratory analyses suggested that situational IU partially mediated this effect on indecisiveness. More details are provided in the Discussion. This finding provides evidence that reducing IU may help improve indecisiveness.

Notably, participants in that study were not preselected for particular levels of IU or indecisiveness. However, IU and indecisiveness are particularly relevant for people with mental disorders. More specifically, IU is considered a transdiagnostic risk factor for mental disorders (McEvoy and Erceg‐Hurn 2016). Elevated IU levels are found across a range of mental disorders (Carleton et al. 2012), and IU levels correlate with the symptom severity of various disorders (McEvoy et al. 2019). Approaches have been developed to reduce IU both for the treatment of specific diagnoses (Hebert and Dugas 2019) and across diagnoses (Mofrad et al. 2020), and these approaches appear to be similarly effective as established forms of therapy (Miller and McGuire 2023). Likewise, indecisiveness is associated with several mental disorder symptoms, like obsessive‐compulsive symptoms (e.g., Frost and Shows 1993), or anxiety disorder symptoms (Rassin and Muris 2005b).

Investigating the causal relationship between IU and indecisiveness is therefore relevant for understanding mechanisms of mental disorders. If the causal effect of IU on indecisiveness applies to individuals with mental disorders as well, this would corroborate the study of Appel and Gerlach (2025a) and extend the validity of their findings to more pronounced forms of indecisiveness in clinical populations. Moreover, reducing IU could offer a promising therapeutic strategy for addressing indecisiveness.

Note that indecisiveness is a consequential associated feature of mental disorders for several reasons. The pervasiveness of decisions in everyday life implies that indecisiveness can cause general difficulties, like putting off important decisions (Ferrari 1994; Germeijs and De Boeck 2002). Also, indecisiveness may lead to disorder‐specific complications, thus contributing to the maintenance of the disorder. A socially anxious person, for instance, might procrastinate deciding to accept or decline a new management position at work until their supervisor gets angry, causing more social anxiety. Given this particular relevance of indecisiveness for mental disorders, the current study tested the causal effect of IU on indecisiveness in a clinical sample.

Building on and extending the results by Appel and Gerlach (2025a), we used the same experimental IU manipulation to replicate the causal role of IU for indecisiveness. We also measured situational IU (cf. Appel and Gerlach 2025a). Using this measure, we examined whether the manipulation effectively changed IU and whether this, in turn, impacted indecisiveness.

More specifically, we predicted that an experimental increase in IU would result in higher (situational) IU levels compared to an experimental reduction of IU (H1). Regarding the implications for decision making, we tested the hypothesis that experimentally increasing IU would lead to higher indecisiveness levels relative to experimentally reducing IU (H2). We also hypothesized that situational IU would mediate the effect of the manipulation on indecisiveness (H3), supporting the assumed mechanism that IU drives changes in indecisiveness. We tested these predictions in a sample of individuals with anxiety disorders and/or OCD. These diagnoses were included for two reasons. First, anxiety disorders and OCD have considerable overlap with each other (Storch et al. 2008): For example, both involve anxiety‐inducing fears and avoidant or safety‐seeking compensating behavior as defining characteristics. Second, both anxiety disorders (Carleton et al. 2012) and OCD (Tolin et al. 2003) seem to have a particularly close connection to IU (Miller and McGuire 2023).

## 2 Methods

The present study was conducted as part of a larger project that involved the same sample and included multiple sub‐projects, each addressing distinct research questions. The hypotheses and analysis plan specific to this study (https://aspredicted.org/6ykr_ybr4.pdf) were preregistered independently of the overall design of the broader project (osf.io/kuj9n). The preregistration for the overarching project also details all variables and procedures. Ethical approval for the project was granted by the ethics committee of the Faculty of Humanities at the University of Cologne (November 28th, 2022; reference no. HAHF0171). All data and analysis scripts needed to replicate the findings are available in the online repository (osf.io/k8fhu), alongside additional materials and analyses. Minor deviations from the preregistration were necessary (e.g., using R instead of SPSS for analyses), and these are thoroughly detailed in the online repository.

### 2.1 Power Analysis and Participants

Pre‐registered power analyses determined that a sample size of *N* = 158 was required to achieve 80% power. Due to limited resources, we preregistered an interim analysis (da Silva Frost and Ledgerwood 2020) after collecting 50% of the target sample (details provided in the online repository). Data collection would stop (a) if the smaller sample already provided sufficient evidence for the hypotheses or (b) if futility analyses—based on Bayes factors—indicated a very low likelihood of finding support for the hypotheses. As neither condition was met, data collection continued. The alpha level was adjusted to account for the interim analysis (see Section 2.4).

Participants were recruited via the crowdsourcing platform Prolific.co, online self‐help forums, social media, and student mailing lists. They were compensated with 0.80 £/1 € for completing the screening, 8 £/9 € for participating in the interview, and 5.20 £/6 € for the main study. Eligibility criteria included being 18 years or older, residing in Germany, and meeting diagnostic criteria for obsessive‐compulsive disorder (OCD) and/or at least one of the following anxiety disorders: panic disorder (PD), agoraphobia (AP), illness anxiety disorder (IAD), social anxiety disorder (SAD), or generalized anxiety disorder (GAD). Major depressive disorder (MDD), a common comorbidity (e.g., Hirschfeld 2001), was also assessed. Exclusion criteria included acute psychosis or imminent risk of harm to self or others, although no participants in the current sample met these conditions.

Out of the 457 individuals who completed the screening, *N* = 250 participated in the subsequent interview. From this group, *N* = 183 fulfilled diagnostic criteria for at least one of the targeted disorders and successfully completed all components of the overarching project. For the present study, *N* = 154 participants met the eligibility criteria and were included in the final sample (mean age: 28.8 years, SD = 7.1) for the analyses conducted below. Exclusions were based on the following pre‐registered criteria (with some participants meeting multiple criteria): being under the age of 18 (*n* = 1), being mistakenly included despite not meeting the diagnostic criteria (*n* = 1), not being assigned to an experimental condition due to a programming error (*n* = 5), failing the attention check (*n* = 9), incorrectly answering critical memory questions about the study instructions (*n* = 4), answering more than 60% of all memory questions incorrectly (*n* = 1), failing to produce a valid free text entry in the experimental manipulation (*n* = 16), or during the indecisiveness measurement (*n* = 6). To assess the last two criteria, two independent raters evaluated all free text entries to confirm that they contained the required content. Agreement between raters was 97.3% for texts pertaining to the experimental manipulation and 93.4% for texts pertaining to the indecisiveness measurement. Disagreements were resolved through discussion. Sample characteristics are summarized in Table 1.

**Table 1:** Sample characteristics.

Variable	Category	*n*	%
Gender	Female	100	64.9
	Male	48	31.2
	Other	5	3.2
	No comment	1	0.6
Age (in years)	18–30	102	66.2
	31–40	42	27.3
	41–50	8	5.2
	51–60	2	1.3
Education	Secondary school, lower level	0	0
	Medium level	13	8.4
	Higher level	62	40.3
	College/university/doctoral degree	79	51.3
Diagnosis	Panic disorder	28	18.2
	Generalized anxiety disorder	69	44.8
	Agoraphobia	21	13.6
	Social anxiety disorder	74	48.1
	Obsessive‐compulsive disorder	27	17.5
	Illness anxiety disorder	31	20.1
	Major depressive disorder	82	53.2
Number of diagnoses per participant (comorbidity)	1	46	29.9
	2	59	38.3
	3	31	20.1
	4	15	9.7
	5	3	1.9
Medication	Currently ongoing	53	34.4
	No current medication	101	65.6
Psychothera‐ peutic treatment	Currently ongoing	67	43.5
	None (but within the last 12 months)	20	13
	None in the last 12 months	67	43.5

*Note:* More than one diagnosis per person could be assigned, so percentages do not add up to 100%; the “Other” category in the Gender item was included to reflect identification outside the binary.

### 2.2 Materials and Measures

#### 2.2.1 Diagnostic Interview

Diagnoses were determined with an interview following the International Diagnostic Checklists for ICD‐10 (IDCL, Hiller et al. 1995). The IDCL comprise semi‐structured interviews based on checklists to systematically assess ICD‑10 diagnostic criteria. Psychometric evaluation studies have demonstrated good reliability comparable to other clinical diagnostic interviews (Hiller et al. 1990, 1995). Given that ICD‑10 criteria for anxiety disorders and OCD largely overlap with DSM‑5 definitions (Craske et al. 2017; Simpson and Reddy 2014), we converted ICD‑10 diagnoses to their DSM‑5 equivalents, as specified in the preregistration. Exclusion criteria were assessed either by direct questioning (imminent risk of harm to self or others) or by clinical judgment based on participants’ statements and behavior during the interview (signs of acute psychosis).

Interviews were conducted by the first author, who holds a PhD in psychology and is a licensed psychotherapist, and by two research assistants who were in their final year of a master's program in clinical psychology and thus had extensive knowledge of the relevant disorders and practical experience in psychological interviewing. The training involved comprehensive instruction on the IDCL, role‐play, and interviews supervised by the first author, including detailed feedback and discussion. All diagnoses relevant to the study were assessed, plus major depressive disorder (MDD).

#### 2.2.2 Intolerance of Uncertainty

To manipulate IU, we relied on a procedure developed by Britton and Davey (2014) translated and adapted for the current context (Appel and Gerlach 2025a). Participants were presented with a fictional scenario involving a woman named Sarah, who described some challenges in her life. They were asked to offer advice to help Sarah. Afterward, they were shown five sample pieces of advice and had the option to revise their own advice if desired.

In the Increase IU condition (*n* = 78), Sarah described herself as unable to respond appropriately to uncertain situations, thus acting recklessly. Participants were asked to provide advice encouraging a stronger reaction to uncertainty (i.e., becoming more *intolerant* of uncertainty). This was supported by 5 items of sample advice, such as: “You need to let yourself feel anxiety, stress, and worry when you are confronted with uncertainty.”

In contrast, in the Decrease IU condition (*n* = 76), Sarah stated she was overly sensitive to uncertainty and thus too cautious. Participants were instructed to offer advice encouraging greater *tolerance* (i.e., less intolerance) of uncertainty, which was also reflected in the sample advice, for example: “Usually, what is uncertain at first turns out to be totally harmless.” (cf., Britton and Davey 2014).

To check whether the manipulation was successful, IU was assessed via three items (Appel et al. 2024) adapted from the German translation (Gerlach et al. 2008) of the Intolerance of Uncertainty Scale (Freeston et al. 1994). The items were as follows: “Uncertainty makes me uneasy, anxious, or stressed”; “It frustrates me not having all the information I need”; “The smallest doubt can stop me from acting.” To reflect situational IU experienced at the moment, the original introduction was slightly adapted to: “To what extent do you agree with the following statements right now at this moment?” Additionally, items were answered using a slider bar ranging from 0 (*strongly disagree*) to 100 (*strongly agree*; Britton and Davey 2014). Items were averaged and internal consistency was adequate, Cronbach's *α* = 0.75.

Because the study was part of a larger project, we were able to make use of trait IU scores for additional analyses scrutinizing the preregistered results post‐hoc (see Table 3). Trait IU was measured using the full 18‐item German version (Gerlach et al. 2008) in its original format, rated on a Likert scale ranging from 1 = *Not at all characteristic of me* to 5 = *Entirely characteristic of me*. Cronbach's alpha in the current sample was excellent (0.90), similar to earlier studies using the scale (Gerlach et al. 2008). A sum score across all items was calculated. Sample mean scores are provided in Table 2. There was no significant difference in trait IU between conditions, *t* (152) = 0.98, *p* = 0.328.

**Table 2:** Descriptive statistics.

Measure	Increase IU (*n* = 78)		Decrease IU (*n* = 76)		Total Sample (*N* = 154)		Correlations (*N* = 154)		
	*M*	SD	*M*	SD	*M*	SD	1	2	3
1. Trait IU (18–90)	64.96	11.05	66.89	13.31	65.92	12.21			
2. Situational IU (0–100)	63.02	20.54	53.84	24.13	58.49	22.78	0.53***		
3. Indecisiveness: lottery decision (11–55)	34.21	9.51	34.26	10.84	34.23	10.15	0.47***	0.44***	
4. Indecisiveness: personal decision (11–55)	44.49	6.88	43.91	8.29	44.20	7.59	0.46***	0.40***	0.55***

*Note:* Mean (*M*) and standard deviation (SD) of situational intolerance of uncertainty (IU) and indecisiveness, and Pearson correlation coefficients.

*** *p* < 0.001.

#### 2.2.3 Decision‐Related Tasks and Indecisiveness Measurement

To measure situational indecisiveness, participants were presented with two tasks relating to real decisions. The purpose of the tasks was to provide a decision context to which the indecisiveness measure could be applied. In a pilot study (*N* = 264; mean age = 32.2 years, SD = 11.2; 51.1% male, 47.3% female, 1.5% other), both tasks produced adequately distributed indecisiveness scores.

For the first task, participants saw four lotteries with different prize amounts and probabilities of winning (4 £/4.50 € at 40%, 5.30 £/6 € at 30%, 8 £/9 € at 20%, 16 £/18 € at 10%), with the expected value (prize × probability) held constant. To activate a decision context, they were informed they would choose one of the four lotteries and that these lotteries would actually be paid out at the specified probabilities, giving the decision real‐life consequences. But before choosing, situational indecisiveness was assessed referring to the upcoming decision.

A translated adaptation (Appel et al. 2024) of the shortened 11‐item version (Rassin et al. 2007) of the Indecisiveness Scale (Frost and Shows 1993) was used. The items were rephrased to refer to the specific upcoming decision (e.g., “I become anxious when making *this* decision,” italics added; Appel and Gerlach 2021). Participants responded to each item on a Likert‐type scale from 1 (*strongly disagree*) to 5 (*strongly agree*), with the items summed to create a total score.

Second, they were asked to describe a significant personal decision they were currently facing but had not yet made. To prompt personally relevant descriptions, examples such as “Should I change jobs?” were provided. Focusing on pending rather than completed decisions was chosen to ensure that the decision was current and salient, as opposed to distant or no longer easily relatable past decisions. Participants were required to spend at least 30 seconds writing before moving on. Again, referring to this personal pending decision, they answered the adapted Indecisiveness Scale items. Cronbach's *α* was 0.87 for the personal decision and 0.91 for the lottery decision.

Note that in both cases, situational indecisiveness was assessed prior to the actual decision, as also reflected in the item wording (see example above). In the lottery task, participants subsequently did make a decision within the study while being aware that the lottery would really be carried out, although the actual decision was not the study's focus. On the other hand, for the personally relevant decision, the actual taking of the decision within the study was not possible, given its real‐life and ongoing nature.

### 2.3 Procedure

**Figure 1:**
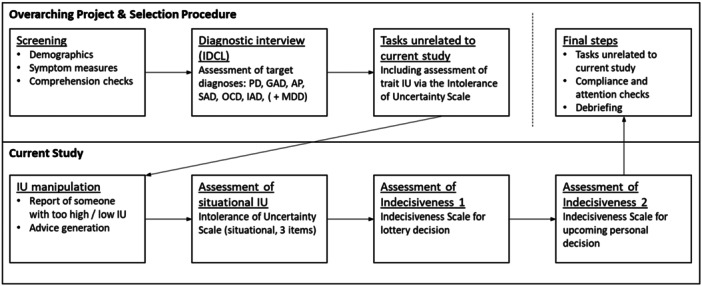
Study flowchart. AP, Agoraphobia; GAD, Generalized anxiety disorder; IAD, Illness anxiety disorder; IDCL, International Diagnostic Checklists for ICD‐10; IU, Intolerance of uncertainty; MDD, Major depressive disorder; OCD, Obsessive–compulsive disorder; PD, Panic disorder; SAD, Social anxiety disorder.

Figure 1 illustrates the procedure. Data collection took place between December 2022 and January 2024. The process began with an initial screening, where participants answered demographic questions and provided information about their symptoms related to OCD and various anxiety disorders. They also completed a questionnaire about COVID‐19‐related safety behaviors, which is not relevant to the present study and did not serve for screening. Participants were invited to continue and select a diagnostic interview appointment if (a) they showed elevated symptom levels for at least one of the required disorders and (b) successfully answered a simple instruction comprehension question.

The diagnostic interview was usually scheduled 2 to 7 days after the screening and was conducted via a video call platform. Participants were eligible for the main study if the interview confirmed at least one of the target diagnoses (excluding MDD, which was assessed only for sample description purposes due to its high comorbidity rate). Following the interview, participants were provided with detailed feedback on their diagnoses and potential treatment options, before either proceeding if a relevant diagnosis was present, or else being thanked and debriefed. Eligible participants were immediately redirected to the main study. Independent of the current study, participants completed two other tasks referring to separate research questions, as well as the trait IU measurement (see Figure 1). All information about these tasks can be found in the preregistration of the overarching project design (osf.io/kuj9n).

Regarding the current study, participants first completed the IU manipulation. They read Sarah's report, generated advice for her, reviewed the sample advice, and revised their own advice if desired. Immediately afterward, the three items measuring situational IU were administered. Following this, situational indecisiveness was measured: First, the lottery task was introduced. After viewing the four lottery options, but before making their choice, participants completed the Indecisiveness Scale referring to the upcoming lottery decision. Next, participants described the important personal decision they were currently facing and indicated their situational indecisiveness in relation to this pending decision. They used the same Indecisiveness Scale items, with the only difference being an additional attention check embedded within the items.

At the conclusion of the study, participants answered questions designed to assess their understanding of the instructions and their compliance with the study procedures. Of these questions, six were specific to the current study, including whether they believed the lottery decision was actually carried out. Participants also rated the level of distraction they experienced during the study on a scale from 1 (*not at all*) to 5 (*strongly*) and indicated whether they recommended the use of their data in the analysis (*yes*/*no*). Additionally, a free‐text field was provided for general comments (none of these raised concerns about data quality). Participants were fully debriefed and given contact information in case they experienced any distress related to the study. All components of the study, except for the interview, were administered via Qualtrics©.

### 2.4 Statistical Analyses

All analyses were conducted in R (R Core Team 2022). To test the hypothesis that experimentally increasing IU results in higher situational IU compared to an experimental reduction of IU (H1), we conducted a *t*‐test for independent samples. To test whether the experimental increase in IU leads to higher indecisiveness levels regarding the two decision situations (H2), we computed a multivariate analysis of variance (MANOVA). Finally, we computed a path analysis examining whether situational IU mediates the effect of the experimental condition (Increase vs. Decrease IU) on situational indecisiveness (H3).

**Figure 2:**
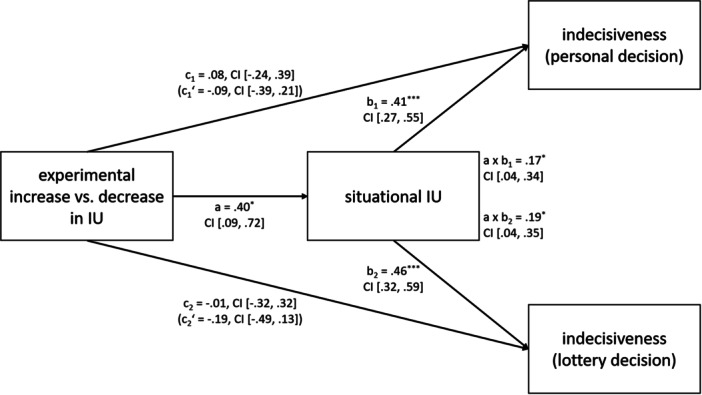
Results of the mediation model. *Note:* CI = 95% confidence interval; path coefficients are standardized. *p < 0.05, ***p < 0.001.

The path analysis was conducted using the *lavaan* package in R (Rosseel 2012). The experimental increase versus decrease of IU served as the key predictor in the path model. The scores of situational indecisiveness in the personal and the lottery decision served as criteria. And the situational IU score was entered as the mediator into the model. Figure 2 illustrates the model. The model was fitted with a maximum likelihood approach and standard errors were computed using bootstrapping with 5000 samples. The total indirect effect of experimentally induced IU on indecisiveness via situational IU was quantified as the sum of both indirect effects in the mediation model:

$$\beta_{\mathrm{indirect}}=\beta_{a}\cdot \beta_{b1}+\beta_{a}\cdot \beta_{b2}\;$$

where *β*_a_ is the regression coefficient of the experimentally induced IU on situational IU, *β*_b1_ is the regression coefficient of situational IU on indecisiveness in the personal decision, and *β*_b2_ is the regression coefficient of situational IU on indecisiveness in the lottery decision.

To assess the robustness of our results, we conducted additional analyses excluding participants who did not believe the lottery decision was actually carried out (*n* = 9) and excluding participants who had indicated concerns over the usability of their own data (*n* = 4). The findings of these (preregistered) robustness analyses remained consistent to the findings of the main analyses that were based on the complete sample (i.e., without the exclusions listed above) and that will be reported in the following results section. All details can be found in the online repository.

## 3 Results

The descriptive statistics are displayed in Table 2. We found that the experimental manipulation of IU was successful (H1). Participants in the Increase condition showed significantly higher values of situational IU (*M* = 63.02, SD = 20.54) than participants in the Decrease condition (*M* = 53.84, SD = 24.13), *t*(146.94) = 2.54, *p* = 0.012, *d* = 0.41, 95% CI [0.09, 0.73]. Contrary to H2, however, a MANOVA did not yield a significant effect of the experimental IU manipulation on indecisiveness in the personal decision and in the lottery decision, *F*(2, 151) = 0.17, *p* = 0.841. The experienced indecisiveness in these decision situations seemed to be unaffected by the experimental manipulation of IU.

Although the MANOVA did not yield a total effect of experimentally manipulated IU on indecisiveness, we computed a path analysis to investigate whether the experimental manipulation of IU had an indirect effect on indecisiveness via situational IU. In other words, we examined the mediating effect of situational IU on the relationship between experimentally manipulated IU and indecisiveness (see Figure 2). Corroborating the findings of the MANOVA for H2, the path analysis did not yield a significant total effect of experimentally manipulated IU on indecisiveness, neither for the personal decision, *β* = 0.08, SE = 0.16, *z* = 0.48, *p* = 0.634, nor for the lottery decision, *β* = −0.01, SE = 0.16, *z* = −0.04, *p* = 0.972. However, the experimental manipulation of IU had a significant effect on situational IU, *β* = 0.40, SE = 0.16, *z* = 2.54, *p* = 0.011, replicating the results of the *t*‐test on H1. Furthermore, more situational IU was significantly linked to higher levels of indecisiveness in both the personal decision, *β* = 0.41, SE = 0.08, *z* = 5.03, *p* < 0.001, and the lottery decision, *β* = 0.46, SE = 0.07, *z* = 6.54, *p* < 0.001. Most importantly, the path analysis showed that situational IU had a significant mediating effect on the relationship(s) between the experimental IU manipulation and both measures of indecisiveness (total mediating effect: *β* = 0.35, SE = 0.15, *z* = 2.35, *p* = 0.019; mediating effect on indecisiveness in the personal decision: *β* = 0.17, SE = 0.08, *z* = 2.19, *p* = 0.028; mediating effect on indecisiveness in the lottery decision: *β* = 0.19, SE = 0.08, *z* = 2.37, *p* = 0.018). In other words, the path analysis revealed that despite the lack of a total and direct effect of experimentally manipulated IU on indecisiveness, there was a clear indirect effect via situational IU.

To rule out that the effects in the path model are driven by trait IU (instead of the experimental manipulation of IU), we extended the preregistered analyses and computed a second path model in which we controlled for the impact of trait IU on the mediator (situational IU) and on the criterion variables (indecisiveness in the personal and lottery decisions). This path model yielded the same pattern of results, indicating that trait IU did not confound the observed associations. These results also highlight that the effects of situational IU are unique to situational IU and cannot be explained by trait IU. The full results are displayed in Table 3.

**Table 3:** Results of the path analysis controlling for trait IU.

*Regressands* and regressors	*β*	SE	*z*	*p*
**Relationships with trait IU**				
*Situational IU*	0.55	0.06	8.62	< 0.001
*Indecisiveness (personal)*	0.35	0.10	3.66	< 0.001
*Indecisiveness (lottery)*	0.32	0.09	3.60	< 0.001
**Mediation model**				
*Situational IU*				
Condition: Increase versus Decrease	0.49	0.13	3.72	<0.001
*Indecisiveness (personal)*				
Condition: Increase versus Decrease	0.05	0.15	0.32	0.747
Situational IU	0.21	0.09	2.28	0.023
*Indecisiveness (lottery)*				
Condition: Increase versus Decrease	−0.07	0.15	−0.45	0.651
Situational IU	0.28	0.09	3.23	0.001
**Indirect effects**				
On indecisiveness (personal)	0.10	0.06	1.81	0.071
On indecisiveness (lottery)	0.14	0.06	2.31	0.021
On indecisiveness (personal + lottery)	0.24	0.11	2.28	0.023

*Note:* IU = Intolerance of uncertainty. The results are grouped by the path coefficients (*β*) indicating the impact of IU (“Relationships with IU”), the path coefficients of the actual mediation model (“Mediation model”) and the calculated path coefficients of the indirect effect(s) (“Indirect effects”). Regressands are printed in italics, regressors are printed in non‐italics.

Abbreviations: SE, standard error of the path coefficients.

Additionally, to rule out potential effects of depressiveness given its relatively high prevalence in the sample, we exploratorily repeated the preregistered main analyses adding MDD severity according to DSM‐5 (no MDD, mild, moderate, severe) as a covariate. Results remained unchanged. Details are provided in the online repository.

## 4 Discussion

In this study, an experimental manipulation of IU was used to assess its causal effect on indecisiveness in two real decisions. The first notable finding is that we observed more IU in the Increase IU compared to the Decrease IU condition, indicating a successful manipulation. Most importantly, while the IU manipulation did not directly affect indecisiveness, it had a significant indirect effect via situational IU. There were no trait IU differences between the experimental conditions that can explain these findings, and exploratory analyses showed that the indirect effect was robust to controlling for trait IU and depression severity.

The successful manipulation of IU is in itself a relevant finding. It indicates that even in a sample diagnosed with disorders linked to high IU, IU levels can be temporarily influenced (i.e., may situationally increase or decrease). However, compared to earlier studies employing this experimental IU manipulation (Appel and Gerlach 2025a; Britton and Davey 2014), the observed effect was smaller, possibly indicating that IU levels in clinical samples are less susceptible to variation.

The indirect effect of the manipulation on indecisiveness mediated by situational IU aligns well with the pathway predicted in H3: The IU manipulation temporarily leads to more or less IU, which in turn is linked to more/less indecisiveness. These findings partly replicate those of Appel and Gerlach (2025a), who found a causal effect of experimentally manipulated IU on indecisiveness. The current study provides initial evidence that this effect extends to a clinical sample characterized by high (trait) IU. However, comparing the study results also reveals differences: As opposed to the significant main effect (i.e., total effect) of the IU manipulation on indecisiveness in the study by Appel and Gerlach (2025a), there was no total effect in the current study, only an indirect effect via situational IU. Although this pattern may appear counterintuitive at first, contemporary mediation approaches do not require a significant total effect for an indirect effect to be meaningful (Hayes 2009; Zhao et al. 2010).

On a theoretical level, our findings are perhaps best explained by elevated levels of indecisiveness and IU in our sample (see Table 2) compared to those observed by Appel and Gerlach (2025a). The descriptives from their study are displayed in Table 4. These higher indecisiveness levels in the current *clinical* sample converge with existing evidence that indecisiveness is related to symptoms of various anxiety‐related disorders (for an overview, see Lauderdale et al. 2024), and is elevated in clinical as opposed to non‐clinical samples (McCabe‐Bennett et al. 2025).

**Table 4:** Descriptive statistics of main variables in Appel and Gerlach (2025a).

Measure	Increase IU (*n* = 101)		Decrease IU (*n* = 101)		Control (*n* = 99)		Total sample (*N* = 301)	
	*M*	SD	*M*	SD	*M*	SD	*M*	SD
Situational IU (0–100)	56.60	18.30	46.74	23.91	46.54	24.45	49.98	22.80
Indecisiveness: lottery decision (11–55)	22.66	7.79	18.70	6.95	21.07	7.42	20.81	7.55
Indecisiveness: personal decision (11–55)	36.29	8.34	34.35	8.67	36.67	7.36	35.76	8.18

*Note:* Mean (*M*) and standard deviation (SD) of situational intolerance of uncertainty (IU) and indecisiveness.

Due to these elevated baseline levels of indecisiveness, the IU manipulation may be less likely to produce a change in distal outcomes like indecisiveness. In contrast, situational IU is a proximal outcome of the IU manipulation because the experimental manipulation targets precisely what is measured with the situational IU items. This conceptual proximity of predictor and outcome variable makes changes in the outcome variable more likely to be detected despite elevated baseline levels.

The significant indirect effect then indicates that the mechanism that explains the causal effect of the IU manipulation on indecisiveness in a non‐clinical population (Appel and Gerlach 2025a) is also present in a clinical population. In other words, the mediation model suggests that changes in situational IU induced by the manipulation explained changes in indecisiveness, even though the total effect of the manipulation on indecisiveness may not have been detectable due to limited statistical power.

Simulation studies have demonstrated that, in mediation analyses, the indirect effect test can have more statistical power than the total effect test (Rucker et al. 2011). In such cases, Loeys et al. (2015) recommend sensitivity analyses controlling for possible confounds to rule out that an indirect effect in the absence of a total effect is spurious. In this sense, the exploratory additional mediation analyses controlling for trait IU and depressiveness are consistent with a robust pattern, making these analyses an important complement to Appel and Gerlach (2025a). Future studies could also include measures of trait indecisiveness to account for baseline levels in statistical analyses.

That said, the main effect of the experimental manipulation reported by Appel and Gerlach (2025a) in a non‐clinical sample lends greater plausibility to a causal interpretation of their findings. Given that no such effect occurred in our clinical sample and that only an indirect effect via the mediation path was observed, inferences about the causal influence of the manipulation on indecisiveness should remain tentative.

Several methodological strengths of this study underline the importance of the findings. Most importantly, the use of a large, carefully selected sample formally diagnosed with mental disorders linked to high IU allows conclusions about clinically relevant levels of IU and indecisiveness. The fact that the study was preregistered and sample size was based on a thorough power analysis supports the reliability of the findings. The close adherence to the method of Appel and Gerlach (2025a) moreover warrants the comparability of results and thus contributes important insights on the effect of IU on indecisiveness. Also, real decisions were included, assuring ecological validity. Furthermore, rigorous and preregistered data quality checks were implemented and sensitivity analyses verified that the findings were robust to unusual cases like those who did not believe the lottery decision was real.

A number of limitations must also be considered. First, while collecting a clinical sample certainly implies strong methodology, the sample was relatively heterogeneous in terms of diagnoses, comorbidity, and current treatment status. This was partly for pragmatic reasons to ensure a sufficiently large sample. However, the disorders are linked by conceptual similarities and their relation to IU, ensuring their relevance to the research question. Also, this approach may allow for interesting follow‐up analyses, such as subgroup comparisons and covariate inclusion, since the data are publicly available for future scientific use.

The nature of the IU manipulation might be another limitation. To ensure a sufficient sample size (i.e., statistical power) despite limited resources, we implemented only two of the three experimental conditions described by Appel and Gerlach (2025a), omitting the neutral control condition. This prevents us from comparing the experimental conditions to a neutral baseline and from determining whether the observed effects were primarily driven by increases or decreases in IU.

Moreover, this type of manipulation is not likely to have a lasting impact because it was very specific and referred to a case report that was obviously fictitious. Although an established and effective approach, it primarily relies on processes typically used in cognitive restructuring (Beck et al. 1979), such as perspective shifting (advising the protagonist) and cognitive reappraisal (e.g., arguing why uncertainty can be good). As such, it does not encompass the full range of mechanisms that have been shown to be effective in the therapeutic modification of IU, including, for example, mindfulness‐related mechanisms, such as acceptance‐based approaches (Morriss 2025). This limited scope may help explain the relatively small effect sizes observed (*d* = 0.41). In a similar vein, IU levels were above the scale midpoint even in the Decrease condition (see Table 2), as opposed to the scores in this condition observed by Appel and Gerlach (see Table 4). While we still observed the predicted relative difference in situational IU between the Increase and Decrease conditions necessary to test our hypothesis, the manipulation did not produce substantial or enduring changes in IU.

In a longitudinal study, Knowles et al. (2022) found that the stable (i.e., trait) components of IU appear to account for the strongest links with psychopathology, which could include indecisiveness in individuals with mental disorders. These components are presumably less responsive to short‐term manipulations. In future studies, more extensive interventions against IU (for an overview, see Miller and McGuire 2023) drawing on more mechanisms for IU change (Morriss 2025) should therefore be examined regarding their impact on indecisiveness.

This being said, the results have important implications. On a more theoretical level, they provide further evidence for the role of IU as a risk factor for indecisiveness (Rassin 2007), partly replicating the results by Appel and Gerlach (2025a). More generally, this insight also adds to the evidence demonstrating the general relevance of uncertainty and its (in‐) tolerance for decision making (Carleton et al. 2016; Jacoby 2020; Luhmann et al. 2011).

Most importantly, the present study extends these insights to mental disorders that frequently involve high indecisiveness. It offers promising initial evidence suggesting that improving IU in mental disorders may also lead to improvements in indecisiveness. This is important because high indecisiveness is linked to diverse disorder symptoms (Frost and Shows 1993; Koerner et al. 2017; Rassin and Muris 2005a). Accordingly, although not in itself pathological, indecisiveness represents a potentially problematic trait. Moreover, indecisiveness could aggravate the situation of those suffering from disorders because decision‐making plays such an important role in psychological functioning and daily life (e.g., Germeijs and Verschueren 2010). This highlights the need for treatment approaches focused on IU reduction.

Several methods to reduce IU have been proposed, working through different psychological mechanisms (Morriss 2025), including changes in IU‐related cognitions (e.g., Dugas and Robichaud 2007), exposure and inhibitory learning to update threat expectations around uncertainty (e.g., Goldman et al. 2007), and mindfulness‐based processes such as decentering and acceptance (e.g., Mathur et al. 2021). For example, Dugas et al. (2022) used behavioral experiments for individuals with GAD to challenge beliefs that contribute to the maintenance of the disorder, particularly regarding the danger and distress associated with uncertain situations. Overall, results of several RCTs have demonstrated the efficacy of IU‐targeted treatments (Miller and McGuire 2023).

A potential mechanism through which such interventions may exert their effects on both IU and indecisiveness is the reduction of safety behaviors (Dugas et al. 2022; Morriss 2025). For example, Appel and Gerlach (2025b) reported correlational evidence that, in decision‐making contexts, higher IU is associated with increased engagement in uncertainty‐reducing behaviors, such as excessive information seeking. These behaviors resembled safety behaviors observed in anxiety disorders (e.g., overpreparing for social situations in social anxiety disorder). This association was partly accounted for by indecisiveness. Because safety behaviors may maintain distress by preventing corrective learning about uncertainty, their reduction is considered an important mechanism of change across anxiety‐related conditions (Thwaites and Freeston 2005). However, it has not yet been examined whether and how IU‐targeted interventions affect indecisiveness, which remains an important avenue for future research. The present study provides encouraging further support for the notion that a beneficial effect of IU reduction on indecisiveness can be expected. This suggested causal link offers a promising foundation for future research to more directly corroborate this effect.

This being said, although the present study focuses on IU, it represents only one of several predisposing factors contributing to indecisiveness. This is reflected both theoretically (e.g., Rassin 2007), where additional factors such as the tendency to excessively strive for the best possible option (“maximizing”; Schwartz et al. 2002) are proposed, as well as empirically. For instance, Appel and Gerlach (2025a) found that changes in indecisiveness following their experimental IU manipulation were only *partially* explained by changes in measured IU.

Beyond individual differences, contextual factors may also contribute to decision difficulties. For instance, an overabundance of choice—characteristic of many modern societies—has been shown to impair decision‐making (Iyengar and Lepper 2000; for an overview, see Chernev et al. 2015). In addition, social and structural influences may play a role. For example, individuals from ethnic minority backgrounds often feel less involved in participatory decision‐making processes with healthcare providers (Cooper‐Patrick 1999). Such experiences may, over time, affect confidence and engagement in decision‐making. Thus, interventions aiming to reduce indecisiveness in the context of mental disorders, in addition to targeting IU, may also benefit from a holistic perspective, additionally considering contextual and societal factors (Allen et al. 2014).

## 5 Conclusions

The current study demonstrates that, in a sample diagnosed with anxiety disorders and OCD, experimentally manipulating IU has an indirect reinforcing effect on indecisiveness in realistic decisions, mediated via increased situational (“state”) IU. The findings add tentative evidence for a causal role of IU for decision making difficulties as found recently in a non‐clinical sample, extending this to disorders with elevated IU and indecisiveness. Because this effect emerged only indirectly through the mediation model, future studies using adapted methodologies are needed to corroborate causal inferences. Indecisiveness, being a potentially impairing trait found at increased levels across diagnoses may therefore be amenable to interventions targeting IU reduction. Considering the importance of decision making both for everyday functioning and for overcoming mental disorders, the effect of therapeutic IU‐focused interventions on indecisiveness should be further examined in future research.

## Conflicts of Interest

The authors declare no conflicts of interest.

## Declaration of Generative AI and AI‐Assisted Technologies in the Writing Process

During the preparation of this work the authors used ChatGPT in order to assist with wording and formulations. After using this tool/service, the authors reviewed and edited the content as needed and take full responsibility for the content of the publication.

## Data Availability

The data that support the findings of this study are openly available in OSF at https://osf.io/k8fhuos.
